# Three-gene based phylogeny of the Urostyloidea (Protista, Ciliophora, Hypotricha), with notes on classification of some core taxa^☆^

**DOI:** 10.1016/j.ympev.2013.10.005

**Published:** 2014-01

**Authors:** Jie Huang, Zigui Chen, Weibo Song, Helmut Berger

**Affiliations:** a Laboratory of Protozoology, Institute of Evolution & Marine Biodiversity, Ocean University of China, Qingdao 266003, China; b Consulting Engineering Office for Ecology, 5020 Salzburg, Austria

**Keywords:** Ciliophora, Evolution, Three genes, Phylogeny, Urostyloidea

## Abstract

Classifications of the Urostyloidea were mainly based on morphology and morphogenesis. Since molecular phylogeny largely focused on limited sampling using mostly the one-gene information, the incongruence between morphological data and gene sequences have risen. In this work, the three-gene data (SSU-rDNA, ITS1-5.8S-ITS2 and LSU-rDNA) comprising 12 genera in the “core urostyloids” are sequenced, and the phylogenies based on these different markers are compared using maximum-likelihood and Bayesian algorithms and tested by unconstrained and constrained analyses. The molecular phylogeny supports the following conclusions: (1) the monophyly of the core group of Urostyloidea is well supported while the whole Urostyloidea is not monophyletic; (2) *Thigmokeronopsis* and *Apokeronopsis* are clearly separated from the pseudokeronopsids in analyses of all three gene markers, supporting their exclusion from the Pseudokeronopsidae and the inclusion in the Urostylidae; (3) *Diaxonella* and *Apobakuella* should be assigned to the Urostylidae; (4) *Bergeriella*, *Monocoronella* and *Neourostylopsis flavicana* share a most recent common ancestor; (5) all molecular trees support the transfer of *Metaurostylopsis flavicana* to the recently proposed genus *Neourostylopsis*; (6) all molecular phylogenies fail to separate the morphologically well-defined genera *Uroleptopsis* and *Pseudokeronopsis*; and (7) *Arcuseries* gen. nov. containing three distinctly deviating *Anteholosticha* species is established.

## Highlights

• 36 new sequences for three genes are characterized from 20 species (12 genera) in the core group of Urostyloidea.
• More well-supported and reliable nodes are detected in the concatenated topologies.
• Multi-gene phylogenies and morphological features are discussed to improve the understanding of the evolution of urostyloids.
• A new genus *Arcuseries* (type *A. petzi*) is established to contain three distinctly deviating *Anteholosticha* species.

## Graphical abstract

**Figure:**
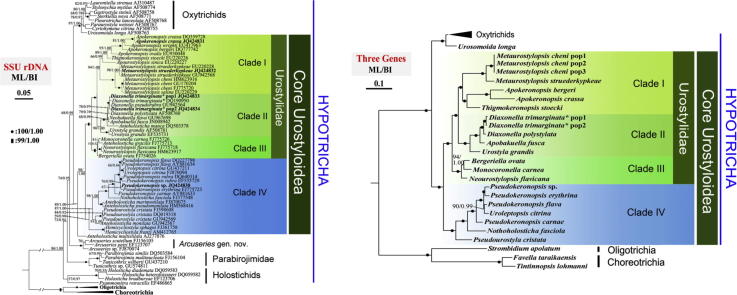

## 1 Introduction

The hypotrichs are a large taxon with many unresolved evolutionary relationships. The phylogeny of several taxa within the subgroup Urostyloidea is little known in part due to the incomplete or inaccurate character states and morphogenetic data. Based on the frontal ciliature and the midventral complex, Berger (2006) grouped most urostyloids into four major taxa (Holostichidae, Bakuellidae, Urostylidae, Epiclintidae) in his review. However, he also pointed that it is not possible to create a usable diagram of the phylogenetic relationships for this group, mainly due to the lack of some important morphogenetic data and the fact that at least one of two main urostyloid features (bicorona or midventral rows) must have evolved convergently. Moreover, the small subunit ribosomal DNA (SSU-rDNA) sequences of many urostyloids are not yet available while new taxa are constantly characterized. Previous molecular studies inferred from the SSU-rDNA locus showed extensive non-monophyly at a number of taxonomic levels (e.g., Foissner and Stoeck, 2011; Paiva et al., 2009; Shao et al., 2011; Wang et al., 2011). This renders the classification of the urostyloids difficult in practice, and our understanding of their evolution remains highly controversial.

Given the uncertainties and conflicts between morphology and the SSU-rRNA gene tree, molecular phylogenies with broader taxon and gene sampling may facilitate us a better understanding of the classification and evolution of the Urostyloidea. Several studies have demonstrated the utility of combined gene trees in inferring phylogenies from SSU-rDNA and LSU-rDNA gene data in different groups of ciliates (Gao et al., 2012; Zhang et al., 2012), providing higher support values and new evidence for some controversial relationships (Huang et al., 2012). Vd’ačný et al. (2012) showed that ITS1 and ITS2 molecules can be used to infer phylogenetic relationships of the Litostomatea not only a species level but also at higher ranks.

In this work, SSU-rDNA, ITS1-5.8S-ITS2 and LSU-rDNA genes for 36 species, 30 of which are characterized for the first time, were sequenced to evaluate the relationships of the clades within the “core Urostyloidea”. The phylogenies inferred from concatenated gene sequences, in consideration of morphological and morphogenetic characters, will facilitate our understanding of the evolutionary history of the urostyloids.

## 2 Materials and methods

### 2.1 Taxon sampling and terminology

The species in this study were selected to represent the morphological and morphogenetic diversity found within the Urostyloidea. Most of the genomic DNA was obtained from previous studies (Table 1).

Five new DNA samples were collected from sites in China (Table 1): *Metaurostylopsis struederkypkeae* from a scallop-farming pond near Laizhou Bay (37°18′N; 119°40′E; April, 2007); *Apokeronopsis crassa* from an intertidal beach near a sewage outfall of No. 1 Beach at Qingdao (36°3′N; 120°20′E; November, 2008); *Diaxonella trimarginata* pop1 and pop2 from a freshwater pond in Zhongshan Park, Qingdao (36°02′N; 120°21′E; summer 2004, 2005; for description of the 2004 population, see Shao et al., 2007b); *Pseudokeronopsis* sp. from Xiaogang, Qingdao (36°4′N; 120°18′E; November, 2007).

**Fig. 1:**
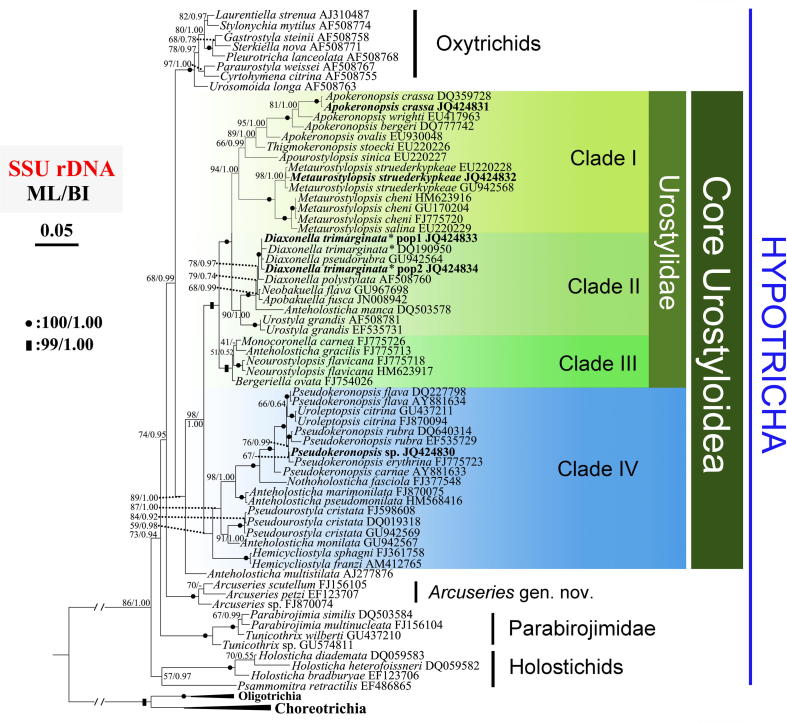
Phylogeny of the Hypotricha inferred by ML of SSU-rDNA sequences. Core urostyloids are labeled in colors. Species newly sequenced in the present study are bold. BP for ML tree and PP for BI tree are given near nodes, respectively. Fully supported (100%/1.00) branches are marked with solid circles. The scale bar corresponds to 10 substitutions per 100 nucleotide positions. The asterisk indicates that *Diaxonella trimarginata* is a junior synonym of *D. pseudorubra* according to Berger (2006). (For interpretation of the references to colour in this figure legend, the reader is referred to the web version of this article.)

**Fig. 2:**
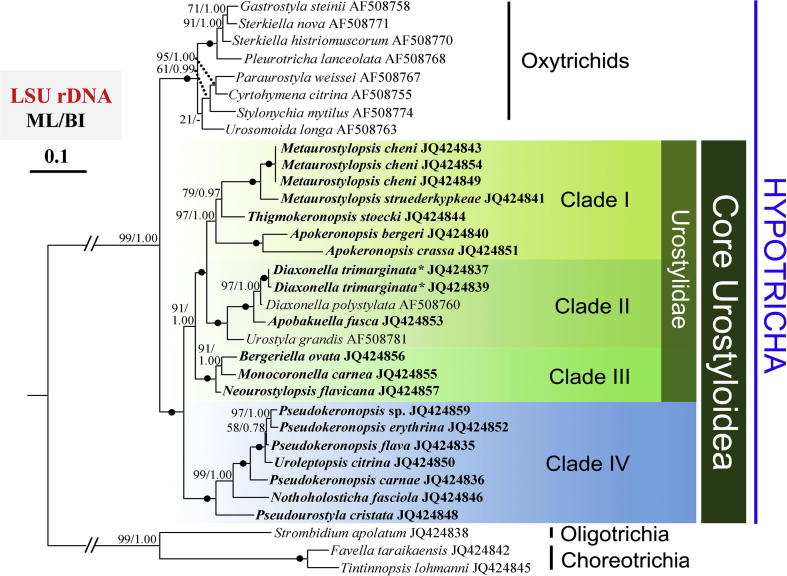
Phylogeny of the Hypotricha inferred by ML of LSU-rDNA sequences. Species newly sequenced in the present study are shown in bold type. BP for ML tree and PP for BI tree are given near nodes, respectively. “−” shows different node topologies between BI and ML trees. Fully supported (100%/1.00) branches are marked with solid circles. The scale bar corresponds to 10 substitutions per 100 nucleotide positions. The asterisk indicates that *Diaxonella trimarginata* is a junior synonym of *D. pseudorubra* according to Berger (2006).

**Fig. 3:**
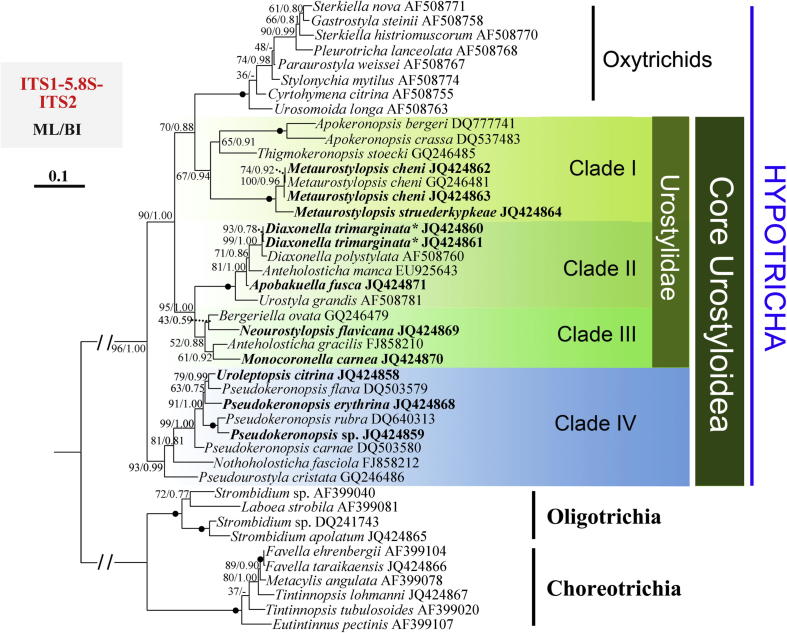
Phylogeny of the Hypotricha inferred by ML of ITS1-5.8S-ITS2 sequences. Species newly sequenced in the present study are shown in bold type. BP for ML tree and PP for BI tree are given near nodes, respectively. “−” shows different node topologies between BI and ML trees. Fully supported (100%/1.00) branches are marked with solid circles. The scale bar corresponds to 10 substitutions per 100 nucleotide positions. The asterisk indicates that *Diaxonella trimarginata* is a junior synonym of *D. pseudorubra* according to Berger (2006).

Ciliates were isolated under a dissecting microscope using glass micropipettes and were identified according to previous descriptions (Shao et al., 2007a,b, 2008a; Song et al., 2006) using live observation and protargol impregnation (Wilbert, 1975). GenBank accession numbers of sequences used in our phylogenetic analyses are listed in Table 1 and Figs. 1–3. Terminology and classification follows Berger (2006) and Lynn (2008), except where noted.

### 2.2 DNA amplification and sequencing

Genomic DNA was extracted using the REDExtract-N-Amp Tissue PCR Kit (Sigma, St. Louis, MO, USA) following the manufacturer’s instructions, with the modification that one tenth of the volume suggested for each reagent solution was used (Gong et al., 2009). Eukaryotic universal 5′ and 3′ primers (Medlin et al., 1988) were used for amplification of SSU-rDNA. A fragment of approximately 500 bp – containing the ITS1, 5.8S ribosomal gene, and ITS2 – was amplified using primers from Yi et al. (2009). A fragment of approximately 1800 bp comprising part of the LSU-rDNA sequences was amplified using primers 28S-1F and 28S-3R from Moreira et al. (2007). Cycling parameters were as follows: 5 min initial denaturation (95 °C), 40 cycles of 15 s at 95 °C, 1 min at 58 °C, and 2 min at 72 °C, with a final extension of 7 min at 72 °C. For some DNA samples, the fragments containing ITS1-5.8S-ITS2 and partial LSU-rDNA were amplified by using the primers ITSF and 28S-3R with the following cycling conditions: 5 min initial denaturation (95 °C), 40 cycles of 15 s at 95 °C, 1 min at 58 °C, and 2 min 30 s at 72 °C, with a final extension of 7 min at 72 °C.

The direct sequencing of PCR products was made preferentially to the cloning. When the sequencing of PCR products failed, they were cloned into the pMD™18-T vector (Takara Biotechnology, Dalian Co., Ltd.) and transformed into the competent *Escherichia coli* strain. Both strands of clones were sequenced on an ABI-PRISM 3730 automatic sequencer (Applied Biosystems). When possible, SSU-rDNA, ITS1-5.8S-ITS2, LSU-rDNA were from the same DNA source. For GenBank accession numbers of newly obtained sequences see Table 1.

### 2.3 Alignments

Four multiple sequence alignments were created for the subsequent phylogenetic analyses, and representatives in the Choreotrichia and Oligotrichia were used as outgroups for all analyses: (1) SSU-rDNA sequences of all available urostyloids plus representatives from non-urostyloid hypotrichs representing a broad taxonomic sample across the Hypotricha (74 sequences in total); (2) ITS1-5.8S-ITS2 region sequences of all available urostyloids plus several oxytrichids (43 sequences in total); (3) LSU-rDNA sequences including all available urostyloid sequences plus some oxytrichids (33 sequences in total); and (4) concatenated sequences of the above three genes for taxa in dataset 3 (33 sequences in total).

Sequences were aligned with MUSCLE v3.7 (Edgar, 2004) with default parameters. Resulting alignments were refined by trimming the sequences on both ends and further edited by eye using BioEdit 7.0.5.2 (Hall, 1999). The LSU-rDNA sequence alignment in dataset 3 was 1836 positions in length, except for oxytrichids which were 1337 bp to 1340 bp. The absent positions at the 3′-end of the LSU-rDNA sequences were treated as missing data as well as in the dataset 4.

### 2.4 Phylogenetic analyses

Bayesian inference (BI) was performed with MrBayes v3.1.2 (Ronquist and Huelsenbeck, 2003) using the GTR + I + G model as selected by AIC in MrModeltest v.2.0 (Nylander, 2004). Bayesian analyses were run twice, each 1,000,000 generations and sampled every 100th. The first 25% of sampled trees were discarded as burn-in prior to constructing a 50% majority rule consensus trees. Maximum likelihood (ML) trees were constructed using RAxML-HPC BlackBox v7.2.7 with optimized parameters on the CIPRES Science Gateway (Stamatakis, 2006; Stamatakis et al., 2008).

### 2.5 Topology testing

In addition to the best ML tree, 16 unrooted trees with enforced topological constraints (Table 2) were built in PAUP, using ML criterion and heuristic search with the tree bisection-reconnection (TBR) and 10 random sequence addition replicates. For all constraints, internal relationships within the constrained groups were unspecified, and relationships among the remaining taxa were unspecified as well. The site-wise likelihoods for the best unconstrained ML tree and all constrained trees were calculated in PAUP under the GTR + I + G model with parameters as suggested by MrModeltest. The reliability of the constrained trees was analyzed in likelihood frameworks through the approximately unbiased (AU) test (Shimodaira, 2002) implemented in the CONSEL software package (Shimodaira and Hasegawa, 2001).

## 3 Results

### 3.1 SSU-rDNA topology (Fig. 1)

Both Bayesian (BI) and maximum likelihood (ML) trees show nearly identical topologies; we cite the ML tree with node supports from both algorithms. As shown in Fig. 1, species in the genus *Anteholosticha* distantly distributed in the dendrograms and formed at least 6 clades. The group composed of *Anteholosticha scutellum*, *A. petzi* and *A.* sp., instead of clustering within the “core Urostyloida”, branched off near the base of the tree. A well-supported cluster of seven representatives of oxytrichids, including five stylonychines, formed a sister to the remaining urostyloids (ML 89%, BI 1.00), of which *Anteholosticha multistilata* branched off firstly. Nevertheless, *A. multistilata* showed ambiguous position in the tree topologies when the SSU-rDNA sequences of some non-urostyloid taxa, such as *Bistichella variabilis*, *Orthamphisiella breviseries*, *Gonostomum* spp., *Cotterillia bromelicola*, *Parabistichella variabilis* and *Uroleptoides magnigranulosa*, are included (data not shown). An 86-taxa SSU-rDNA tree (data not shown) located *A. multistilata* within the non-urostyloid clade with long branch, viz., sister to *Uroleptoides* + *Parabistichella*, suggesting that it might be an artifact caused by either an undersampling or the implausible position is due to other phenomena not yet known or considered, for instance mutational saturation. Moreover, the low sequence divergence among these non-urostyloids hampers phylogenetic analyses and makes them very sensitive to species inclusion. Therefore, these non-urostyloid taxa mentioned above were not included in the final phylogenetic analyses. Due to the ambiguous position of *A. multistilata*, we did not consider it as a member of the core group of urostyloids. As previously shown (Yi and Song, 2011), the remaining urostyloids formed a core group including 17 morphologically well-defined genera (Fig. 1). All phylogenetic analyses consistently and strongly support this group designated as core Urostyloidea (ML 98%, BI 1.00). Within it, Bayesian inference and maximum likelihood analyses depicted two super-clades, I-–III vs. IV (Fig. 1).

The first super-clade (ML 99%, BI 1.00) comprised of 12 genera was divided into three well-supported clades (Fig. 1): Clade I, the *Apokeronopsis*–*Metaurostylopsis* clade; Clade II, the *Diaxonella–Urostyla* clade; and Clade III, the *Bergeriella*–*Monocoronella* clade. The internal relationships within Clade I were rather well resolved with the exception of the position of *Apourostylopsis*. The sister relationship between *Apourostylopsis* and *Apokeronopsis + Thigmokeronopsis* was well supported by a posterior probability of 1.00, but only poorly supported by 66% ML bootstrap values. Nevertheless, their sister relationship with *Metaurostylopsis* was highly recovered in both ML and BI trees (ML 94%, BI 1.00). Within Clade II (ML 90%, BI 1.00), *Urostyla grandis* was sister to the fully supported cluster of *Anteholosticha manca*, *Neobakuella*, *Apobakuella* and *Diaxonella*. The five populations of *Diaxonella* formed a monophyletic group (ML 78%, BI 0.97) and were closely related with the two recently established genera *Neobakuella* and *Apobakuella*. Clade III was strongly supported (ML 99%, BI 1.00), while its internal relationships were far from being settled noting the low bootstrap values. The sister relationship of Clade I and II was maximally supported by both ML and BI analyses (ML 100%, BI 1.00).

The second super-clade (Clade IV) comprised three members of *Anteholosticha* and the majority of taxa assigned to the Pseudokeronopsidae and Pseudourostylidae. There was strong Bayesian support for this clade (BI 0.98), although the support from the maximum likelihood analysis was poor (ML 59%). Two members of *Hemicycliostyla* branch off basally in clade IV. However, the sister relationship between *Hemicycliostyla* and *Pseudourostyla*, proposed by Berger (2006), was not rejected by any of the statistical tests applied, indicating that the Pseudourostylidae is a monophyletic lineage as in the morphological classification. The genus *Anteholosticha* failed to form a monophyletic group in all trees (Table 2). The type species of *Anteholosticha*, *A. monilata*, clustered as sister to *Pseudourostyla cristata* with strong support from both methods (ML 91%, BI 1.00). By contrast, two recently described members of *Anteholosticha*, *A. marimonilata* and *A. pseudomonilata*, clustered close to three genera in the Pseudokeronopsidae with strong supports in all trees (ML 98%, BI 1.00). Within the Pseudokeronopsidae clade, *Nothoholosticha* had a relatively long branch, followed by *Uroleptopsis* and *Pseudokeronopsis*. *Uroleptopsis* invariably nested within the *Pseudokeronopsis* radiation, clustering together with *P. flava*, with low node supports (ML 66%, BI 0.64). The possibility that *Uroleptopsis* falls outside of the *Pseudokeronopsis* clade was rejected by AU tests (Table 2).

### 3.2 LSU-rDNA and ITS1-5.8S-ITS2 topologies (Figs. 2 and 3)

We have substantially improved the broad-scale taxonomic sampling of the LSU-rDNA and ITS1-5.8S-ITS2 sequences, providing 39 new sequences, enabling us to include 20 urostyloids (comprising 17 species) on LSU trees for the first time (Fig. 2). Analyses inferred from 33 taxa of LSU-rDNA sequences showed similar topologies with that derived from 74-taxa SSU-rDNA trees. Both BI and ML analyses strongly depict (ML 99%, BI 1.00) the sister relationships of two main groups, the core Urostyloidea and oxytrichids, respectively. Similar as the SSU-rDNA tree topologies, four well-supported clades were observed within the core Urostyloidea (Fig. 2). The internal relationships within Clades I, II and III were all well recovered except for the node of *Thigmokeronopsis*. There was strong Bayesian support for the sister relationship between *Thigmokeronopsis* and *Metaurostylopsis* (BI 0.97), but it was only moderately supported by 79% ML bootstrap values. The LSU-rDNA phylogeny provided full support for Clade IV, with more limited taxon sampling than in the SSU-rDNA analyses. The position of *Uroleptopsis* differed from that in the SSU-rDNA trees which was sister to a clade formed by three *Pseudokeronopsis* spp. and then followed by *P. carnae*. The possibility of separating *Uroleptopsis* from the genus *Pseudokeronopsis* was rejected by both the AU tests. In general, support for each group was stronger in the LSU-rDNA tree than in the SSU-rDNA tree.

Analyses inferred from 43 taxa of ITS1-5.8S-ITS2 gene sequences show similar species inclusion for each clade with that in the 74-taxa SSU-rDNA trees, while the relationships between clades differed (Fig. 3). The major incongruence between ITS1-5.8S-ITS2 gene trees and other topologies was that the sister relationship of oxytrichids and all urostyloids is not recovered. The oxytrichids and *Urosomoida* grouped with Clade I of the core Urostyloidea with moderate support (ML 70%, BI 0.88), which together formed a group that was sister to Clades II + III with high supports (ML 90%, BI 1.00). The close relationships between Clade II and III were stable with strong supports in both analyses (ML 95%, BI 1.00), while the internal nodes received variable support values. Clade IV, containing *Pseudokeronopsis*, *Uroleptopsis*, *Nothoholosticha*, and *Pseudourostyla*, branched off first in the ITS1-5.8S-ITS2 topologies.

### 3.3 Concatenated three-gene analyses (Fig. 4)

The topologies of the combined three-gene trees with 33 sequences were nearly identical to the LSU-rDNA trees, with the sole exception for the position of *Thigmokeronopsis stoecki*. *Thigmokeronopsis* clustered as sister to *Apokeronopsis* with maximal support in the concatenated analyses, whereas it is sister to *Metaurostylopsis* with moderate to strong support in the LSU-rDNA topologies. Overall, the concatenated data provided stronger node supports across the trees than either of single-gene analyses. Almost all nodes within the core Urostyloidea obtained full support in the three-gene trees, which indicates that the concatenation of the three markers could generate a good resolution for the phylogeny of the core Urostyloidea.

### 3.4 Topology testing (Table 2)

Statistical tests carried out on either single gene or three gene concatenation rejected most of morphologically-based genera or higher taxa as monophyletic lineages (Berger, 2006; Lynn, 2008). There were two exceptions: (1) the monophyly of the family Pseudourostylidae was not rejected by the SSU-rDNA dataset (Table 2). However, the monophyly awaits more data to verify as only one sequence is available for the other two genes; (2) the monophyly of *Pseudokeronopsis* based on the ITS1-5.8S-ITS2 dataset was not rejected by the AU and SH tests. The rejection of the species and genus assignment indicates that the current classifications should be adapted based on new data.

## 4 Discussion

**Fig. 4:**
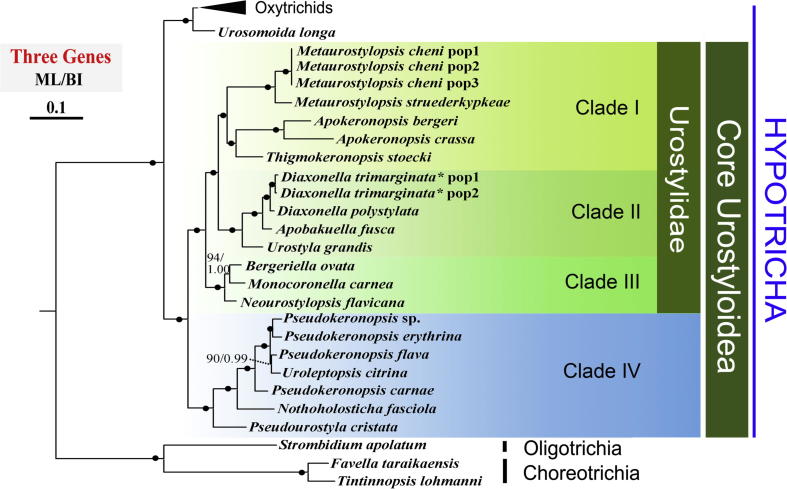
Phylogeny of the Hypotricha inferred by ML of Dataset 4. BP for ML tree and PP for BI tree are given near nodes, respectively. Fully supported (100%/1.00) branches are marked with solid circles. The scale bar corresponds to 10 substitutions per 100 nucleotide positions. The asterisk indicates that *Diaxonella trimarginata* is a junior synonym of *D. pseudorubra* according to Berger (2006).

Previously published and our SSU-rDNA analyses of urostyloids demonstrate as usual, extensive conflicts between the morphological and the molecular data for the main subgroups, namely the Pseudokeronopsidae, the Urostylidae, the Bakuellidae, and the Holostichidae (Berger, 2006). Moreover, SSU-rDNA data do not support some of the morphologically defined genera, i.e., *Pseudokeronopsis* and *Anteholosticha*. Therefore, we applied three-gene analyses to test if additional molecules were also in conflict with the morphologically-based hypotheses of phylogenetic relationships (Figs. 2–4). We also asked if these additional molecular data could provide more resolution in nodes than the SSU-rDNA sequences.

### 4.1 Uroleptopsis citrina and Pseudokeronopsis

According to Berger (2004, 2006), *Uroleptopsis citrina*, the type species of *Uroleptopsis*, differs from *Pseudokeronopsis* species in the transverse cirri (absent vs. present), the position of the buccal cirrus (in line with bicorona vs. in ordinary position, that is, right of paroral), the number of cirri formed from anlage I (two vs. one), the structure of the midventral complex (some anlagen form only one cirrus vs. all anlagen form pairs), and the number of dorsal kineties (three vs. four or more). This rather high number of distinct morphological differences supports the validity of the genus *Uroleptopsis* which was originally established by Kahl (1932). However, this classification was not supported by the SSU-rDNA gene (Huang et al., 2010) as *U. citrina* always clustered in the *Pseudokeronopsis* clade (Fig. 1). Similarly, the non-monophyly of *Pseudokeronopsis* was observed in phylogenies inferred from the other two gene markers (LSU-rDNA and ITS1-5.8S-ITS2) and the concatenated dataset (Figs. 2–4). Additionally, the hypothesis that *U. citrina* could fall outside of the *Pseudokeronopsis* clade was rejected by most topology tests, except for the ITS1-5.8S-ITS2 dataset (*p* = 0.063, Table 2). These molecular results raise the question whether the morphological differences justify the separation of *U. citrina* and *Pseudokeronopsis* species at genus level. Despite the fact that supraspecific categories (e.g., genera, families, orders) cannot be defined objectively (e.g., Ax, 1999), a synonymy of *Uroleptopsis* Kahl, 1932 and *Pseudokeronopsis* Borror and Wicklow, 1983 would entail a large number of nomenclatural acts because many species had to be transferred from the younger synonym *Pseudokeronopsis* to the senior synonym *Uroleptopsis*, a problem already discussed by Berger (2006). Thus, we refrain from this molecular biologically-indicated synonymisation and consider *Uroleptopsis* and *Pseudokeronopsis* as two valid taxa which can be easily separated via morphological features. More sequences in these two taxa (only one in *Uroleptopsis*) are needed to better understand their relationship and gain more insights on the evolutionary pathways of the morphological features.

### 4.2 Thigmokeronopsis and Apokeronopsis

Both *Thigmokeronopsis* and *Apokeronopsis* were placed in the Pseudokeronopsidae because they have a *Pseudokeronopsis*-like bicorona and one marginal row on each side (Berger, 2006; Shao et al., 2007a). However, a close relationship with *Pseudokeronopsis* was never supported in SSU-rDNA phylogenies (Chen et al., 2011a; Huang et al., 2010; Yi et al., 2008a). Accordingly, based on the SSU-rDNA data, Chen et al. (2011a) removed these two genera from the Pseudokeronopsidae and transferred them to the Urostylidae sensu Lynn (2008). In all phylogenies reported here (Figs. 1–4), *Thigmokeronopsis *+ *Apokeronopsis* and *Pseudokeronopsis* were placed in two widely separated clades with strong bootstrap supports (Clade I vs. IV). Moreover, the hypotheses that *Thigmokeronopsis* and *Apokeronopsis* were members of the Pseudokeronopsidae were rejected by all AU tests (Table 2). Interestingly, *Thigmokeronopsis* and *Apokeronopsis* have specific morphological and ontogenetic characteristics in common separating them clearly from *Pseudokeronopsis* and *Nothoholosticha*, namely the cirri of each midventral pair are distinctly separated (vs. ordinarily zigzagging pairs), and the anlagen for the marginal rows and dorsal kineties are formed *de novo* (vs. parental structures contribute; Li et al., 2009; Shao et al., 2007a; Wicklow, 1981; Wirnsberger, 1987). A bicorona is not a very complex characteristic so that a convergent evolution of such a structure in *Pseudokeronopsis* and in *Thigmokeronopsis* + *Apokeronopsis* is conceivable, all the more, as for the oxytrichid *Neokeronopsis* the independent formation of a bicorona is demonstrated (Berger, 2006; Foissner and Stoeck, 2008; Foissner et al., 2010; Warren et al., 2002). Therefore, all molecular results obtained, together with morphological/morphogenetic features mentioned above, strongly suggest that *Thigmokeronopsis* and *Apokeronopsis* should be removed from the Pseudokeronopsidae. Berger (2006) pointed that the prominent thigmotactic cirral field of *Thigmokeronopsis* is an evolutionary novelty, but he did not accept the Thigmokeronopsinae established by Wicklow (1981) because this group was monotypic at that time, that is, contained only the name-bearing type genus. However, the discussion above strongly suggests that *Thigmokeronopsis* and *Apokeronopsis* are closely related and clearly separated from the core pseudokeronopsids, and therefore we re-activate the Thigmokeronopsinae Wicklow, 1981 for this taxon. The topologies of the trees suggest that this group is a member of the Urostylidae to which *Thigmokeronopsis* and *Apokeronopsis* have been assigned just recently by Chen et al. (2011a). According to the SSU-rDNA data, *Apourostylopsis sinica*, type of *Apourostylopsis*, is closely related to *Thigmokeronopsis* and *Apokeronopsis* (Fig. 1). However, this species lacks several features of the thigmokeronopsines (cirri of midventral pairs not distinctly separated; marginal rows not formed *de novo*; bicorona lacking, that is, three frontal cirri) and consequently we do not include it in this taxon (Shao et al., 2008b; Song et al., 2011).

### 4.3 Three-gene phylogenies strongly support the transfer of Metaurostylopsis flavicana into Neourostylopsis

*Neourostylopsis flavicana* was originally assigned to *Metaurostylopsis* by Wang et al. (2011) based on its ciliature. However, in the SSU-rDNA phylogenies by Song et al. (2011) and Wang et al. (2011), it is clearly separated from other species in *Metaurostylopsis*. A more detailed morphological analysis of this species demonstrated that it differs from other *Metaurostylopsis* species by a lower number of frontoterminal cirri (2 vs. 3–8) and the absence of a midventral row, two generic features according to Berger (2006) and Song et al. (2001, 2011). Thus, Chen et al. (2013) established *Neourostylopsis,* comprising *M. flavicana* (type) and *N. orientalis*, with the lack of a midventral row and a midventral complex extending to about mid-body as main features. Our SSU-rDNA analyses (Fig. 1) clearly and strongly support the separation of *Neourostylopsis* from *Metaurostylopsis*. The phylogenies based on the ITS1-5.8S-ITS2 region, the LSU-rDNA, and the combined three-gene trees show the same result (Figs. 2–4). Additionally, the hypothesis that *Metaurostylopsis flavicana* and other species of *Metaurostylopsis* form a monophyletic clade is rejected by AU tests based on all datasets (Table 2). All these molecular results strongly support the transfer of *M. flavicana* to *Neourostylopsis*.

### 4.4 Higher level assignment of Monocoronella, Neourostylopsis, and Bergeriella

*Monocoronella*, at present comprising *M. carnea* (type) and *M. dragescoi*, can be easily distinguished from other urostyloid genera by its unique, well-marked single-rowed corona (Chen et al., 2011b). However, the position of *Monocoronella* is difficult to assess because morphologically similar genera are assigned to different higher taxa by Berger (2006) and Lynn (2008). Due to the unstable SSU-rDNA phylogenies and the contradiction between morphological and molecular data, *Monocoronella* was preliminarily assigned to the Urostylida by Chen et al. (2011b). Our phylogenetic analyses based on SSU-rDNA and ITS1-5.8S-ITS2 data do not indicate convincing and stable relationships as well. *Monocoronella* always falls into a well-supported clade (III) with *Anteholosticha gracilis* (the population gene-sequenced had four frontal cirri, Dr. Jiang, pers. comm.), *Neourostylopsis flavicana* and *Bergeriella ovata*, while the internal relationships within this clade remain unresolved as indicated by low support (Figs. 1 and 2). Nevertheless, phylogenies inferred from LSU-rDNA and three-gene concatenated data (Figs. 3 and 4) strongly support a close relationship of *Monocoronella* and *Bergeriella*. Interestingly, *Neourostylopsis*, *Anteholosticha gracilis* sensu Yi et al. (2010), *Monocoronella*, and *Bergeriella* have more than the ordinary three frontal cirri indicating that the increase in the number of these cirri occurred in the stem-line of the last common ancestor of this group. Of course, the increase in the number of frontal cirri is not a very outstanding feature because it evolved, as has been proven several times independently, in the urostyloids (e.g., pseudokeronopsids, urostylids) and other hypotrichs (*Neokeronopsis*, *Pattersoniella*; for reviews, see Berger, 1999, 2006). Other features, for example, presence of cortical granules, macronuclear apparatus composed of more than two nodules, and lacking caudal cirri, are very likely plesiomorphies at this level. Liu et al. (2010) created the monotypic (and therefore redundant) group Bergeriellidae with the many midventral rows forming a curious pattern in the postoral area as main morphological feature. Since only one species with this type of ciliature is known at present it is too early to decide at which level this feature is an apomorphy. Therefore, this family will likely need to be redefined in the future.

### 4.5 Higher level assignment of Diaxonella and Apobakuella

*Diaxonella* – established by Jankowski (1979) but not assigned to a higher taxon – was classified in the Holostichidae by Oberschmidleitner and Aescht (1996) based on unspecified morphological characters. This assignment was taken over by Berger (2006) mainly because *Diaxonella* has, like *Holosticha*, three frontal cirri and a midventral complex composed of cirral pairs only. He also stated that the formation of the left marginal rows from a common anlage in *Diaxonella* (Jerka-Dziadosz and Janus, 1972; Zhang et al., 1985) has to be interpreted as convergence to *Pseudourostyla* (name-bearing type of the Pseudourostylidae), which has the same (or at least a very similar) mode (e.g., Chen et al., 2010b; Jerka-Dziadosz, 1972). By contrast, Shao et al. (2007b) placed *Diaxonella* in the Pseudourostylidae assuming that this type of left marginal row formation developed only once and because the mode of marginal row formation was regarded as a significant feature at family level by Eigner and Foissner (1992). However, none of these classifications was reflected by molecular phylogenies (e.g., Li et al., 2011; Figs. 1–4) supporting at least the hypothesis by Berger (2006) that this pattern of left marginal row formation evolved twice independently. Li et al. (2011) discussed the various phylogenetic positions of *Diaxonella* based on morphological data and SSU-rDNA sequences. The new phylogenies inferred from LSU-rDNA (Fig. 2), ITS1-5.8S-ITS2 (Fig. 3), and three genes (Fig. 4) support the tree structure derived from SSU-rDNA (Fig. 1), that is, a close relationship of *Diaxonella* and *Urostyla*. This agrees with the classifications by Jankowski (2007) and Lynn (2008) which placed both genera in the Urostylidae, although they differ distinctly in the frontal ciliature: *Urostyla* has many cirri arranged in a bi- or multicorona while *Diaxonella* has the ordinary three frontal cirri which were very likely already present in the immediate common ancestor of the hypotrichs (Berger, 2006, 2008). Assuming that this relationship is correct, a convergent formation of a bicorona in the pseudokeronopsids and other taxa has to be postulated (further details, see Section 4.2).

*Apobakuella* was assigned to the Bakuellidae (for review, see Berger, 2006) because it has three frontal cirri and a midventral complex composed of pairs and rows (Jiang et al., 2013). However, higher taxa of the urostyloids basing on the frontal ciliature and the midventral complex as main features are questioned by molecular data because four genera (*Metaurostylopsis*, *Parabirojimia*, *Neobakuella*, *Apobakuella*) morphologically assigned to the Bakuellidae occupied three clearly separated positions both in SSU and ITS phylogenies and the three-gene topologies in this study (Figs. 1–4). Differing from the assignments by Berger (2006) and Jankowski (2007), Lynn (2008) did not accept the bakuellids and classified them into the synonymy of the urostyloids. However, molecular data of *Bakuella marina* – the type species of the genus *Bakuella* – has not been available yet. Thus, no final comment about the validity of the Bakuellidae can be made out from the molecular data. It is striking that *Apobakuella*, like *Diaxonella* with three frontal cirri, clusters with *Urostyla*. The latter has many, bow-shaped arranged frontal cirri.

### 4.6 Urostyloids paraphyletic according to ITS (Fig. 3)

In the phylogenies based on the ITS1-5.8S-ITS2 region sequences, the urostyloids are not monophyletic because the clade (*Apokeronopsis *+ *Thigmokeronopsis*) + *Metaurostylopsis* is more closely related to the oxytrichids + *Urosomoida* than to the remaining urostyloids. There might be two explanations for this topology: (1) The internal transcribed spacer (ITS) regions, subjecting to higher evolutionary rates than the rRNA gens, are more divergent in terms of both nucleotide sequence and length (Hillis and Dixon, 1991). Accordingly, these regions are considered to be useful for elucidating relationships among congeneric species or populations from different geographic regions (Baldwin, 1992; Yi et al., 2008a; Huang et al., 2011); (2) Considering the limited taxon sampling, the moderate support values, as well as the possibility of the monophyly of urostyloids based on 5.8S-ITS sequences (not rejected by AU test), additional molecular data are required to construct a more reliable topology.

### 4.7 New genus for some deviating Anteholosticha species

*Anteholosticha scutellum* (Cohn, 1866) Berger, 2003, *A. petzi* Shao et al., 2011, and *Anteholosticha* sp. formed a fully supported clade near the base of the SSU-rDNA tree (Fig. 1). These three species and *A. warreni* (Song and Wilbert, 1997) Berger, 2003 have some morphological features in common (see diagnosis) supporting the distinct separation from other groups in the molecular tree. We therefore establish a new genus for these species.

*Arcuseries* gen. nov.

Diagnosis: Marine, non-dorsomarginalian hypotrichs with roughly U-shaped-arranged transverse cirri and midventral complex composed of cirral pairs only. Three frontal cirri, buccal cirrus, frontoterminal cirri, and pretransverse ventral cirri present. One right and one left marginal row. Three bipolar dorsal kineties. Caudal cirri lacking. Undulating membranes roughly straight and more or less arranged in parallel. Many macronuclear nodules. High support by gene sequence data.

Type species: *Arcuseries petzi* (Shao et al., 2011) comb. nov. (Basionym: *Anteholosticha petzi* Shao, Gao, Hu, Al-Rasheid and Warren, 2011.)

Etymology: The genus-group name *Arcuseries* is a composite of the Latin nouns *arcus* (m; bow, bending, curve) and *series* (f; row) alluding to the fact that the transverse cirri of the species included form a curved row. Feminine gender.

Species included: *Arcuseries petzi* (Shao et al., 2011) comb. nov. (basionym *Anteholosticha petzi* Shao et al., 2011). *Arcuseries scutellum* (Cohn, 1866) comb. nov. (basionym *Oxytricha scutellum* Cohn, 1866). *Arcuseries warreni* (Song and Wilbert, 1997) comb. nov. (basionym *Holosticha warreni* Song and Wilbert, 1997).

Remarks: The large genus *Holosticha* Wrzesniowski (1877) was previously a melting pot for all hypotrichs with three frontal cirri, transverse cirri, and a midventral complex composed of zigzagging cirri (Kahl, 1932; Borror, 1972; Borror and Wicklow, 1983). Berger (2003) confined it to species which have, inter alia, a bipartite adoral zone and the anterior end of the left marginal row distinctly curved rightwards (for review, see Berger, 2006). For that reason, most species were removed from *Holosticha*. Most of them were preliminary classified in *Caudiholosticha* Berger, 2003 (with caudal cirri) and *Anteholosticha* Berger, 2003 (without caudal cirri). However, Berger (2003, 2006) already stated that these two genera are heterogeneous due to the lack of morphological apomorphies. Later, the supposed non-monophyly of *Anteholosticha* was supported by molecular analyses (e.g., Shao et al., 2011), including the present one. The species listed above agree, inter alia, in the specific arrangement of the transverse cirri, that is, they form a roughly U-shaped or bow-shaped pattern. By contrast, in *Anteholosticha monilata* (Kahl, 1928) Berger, 2003 – type of *Anteholosticha* – the transverse cirri are arranged in a J-shaped or hook-shaped pattern which occurs also in many other hypotrichs indicating that the U-shape is a derived state. In addition, *A. monilata* has more than three dorsal kineties while the species mentioned above have invariably three kineties which is the plesiomorphic state likely already present in the last common ancestor of the hypotrichs (Berger, 2006, 2008). These morphological differences and the clear separation in the molecular trees are clear evidence that *Anteholosticha petzi*, *A. scutellum*, *A. warreni*, and *Anteholosticha* sp. (GenBank accession number FJ870074) are not congeneric with *A. monilata*. We fix *Anteholosticha petzi* as type of *Arcuseries* because it is defined morphologically, ontogenetically, and molecular biologically from the same population (Shao et al., 2011).

*Arcuseries* is, due to its rather basal branching, sister to a large clade comprising the oxytrichids and the urostylids, at least in the tree shown (Fig. 1). It indicates that the new genus cannot be assigned to an existing higher taxon (e.g., Urostyloidea, Dorsomarginalia) without making it paraphyletic. We therefore preliminarily “classify” *Arcuseries* as non-dorsomarginalian hypotrich, a paraphyletic group comprising basically all hypotrichs (e.g., holostichids, urostylids, parabirojimids, trachelostylids, gonostomatids, amphisiellids), except the dorsomarginaliens, a likely monophyletic group which is characterized by the possession of a dorsomarginal kinety (Berger, 2006). Since the higher-level classification of the hypotrichs is still rather vague, we prefer such a preliminary assignment over the establishment of a further taxon.

Likely there exist some further differences between *Anteholosticha* and *Arcuseries*, for example, in the formation of the adoral zone in the proter (parental zone likely only partially renewed vs. completely renewed) and the formation of the frontal-ventral-transverse cirri anlagen for proter and opisthe (from two separate anlagen vs. via primary primordia; Berger, 2006; Hu et al., 2000; Shao et al., 2011).

*Arcuseries petzi* was described by Shao et al. (2011) while the SSU rRNA gene sequence of this population was deposited by Yi et al. (2008b; as *Anteholosticha* sp.-QD-1) in GenBank with accession number EF123707. By contrast, *Anteholosticha scutellum* (Cohn, 1866) has a rather long history reviewed in detail by Berger (2006). It was redescribed and gene-sequenced by Chen et al. (2010a).

*Anteholosticha* sp. with GenBank accession number FJ870074 (Fig. 1) was named *Anteholosticha parawarreni* by Yi and Song (2011). However, so far the formal description of this species is not yet available and therefore *A. parawarreni* is a nomen nudum. Its description is in preparation and will show that it is very similar to *A. scutellum*, *A. petzi*, and *A. warreni*.

*Anteholosticha eigneri* in Yi and Song (2011), with GenBank accession number GQ258105 (alpha-tubulin gene), also lacks an original description and consequently this name is a nomen nudum too.

*Holosticha warreni* was described by Song and Wilbert (1997) and later transferred to *Anteholosticha* by Berger (2003) because the *Holosticha* apomorphies are lacking (for review, see Berger, 2006). The morphological data and the SSU rRNA gene sequence (Shao et al., 2011) demonstrate that it must be closely related to *A. scutellum* and *A. petzi* and therefore we include *H. warreni* in *Arcuseries*. The cell division does not show peculiarities (Hu et al., 2000).

The marine *Anteholosticha arenicola* (Kahl, 1932) Berger, 2003 is, like *A. scutellum*, rather widely rounded posteriorly and has a slightly curved row of transverse cirri (Berger, 2006). However, since no details about the cirral and dorsal kinety pattern are present we refrain from a transfer to *Arcuseries*. *Anteholosticha thononensis* (Dragesco, 1966) Berger, 2003 has also a similar habitus. However, since the cirral pattern is not described with sufficient accuracy and since it obviously has four dorsal kineties and was isolated from a limnetic habitat a transfer to *Arcuseries* seems not indicated.

*Arcuseries* can be easily separated from other genera with a similar cirral pattern (e.g., *Holosticha*, *Anteholosticha*, *Caudiholosticha*) by the arrangement of the transverse cirri.

## Figures and Tables

**Table 1:** List of species, GenBank numbers, and sequence length (bp) of newly sequenced SSU-rDNA, ITS1-5.8S-ITS2 region, and LSU-rDNA sequences (GenBank numbers for new sequences are in bold).

Species (Reference)	SSU-rDNA		ITS1-5.8S-ITS2		LSU-rDNA	
	Acc. No.	Length	Acc. No.	Length	Acc. No.	Length
*Apobakuella fusca* (1)	JN008942	1766	**JQ424871**	549	**JQ424853**	1855
*Apokeronopsis bergeri* (2)	DQ777742	1765	DQ777741	468	**JQ424840**	1850
***Apokeronopsis crassa*** (3)	**JQ424831**	1765	DQ537483	466	**JQ424851**	1729
*Bergeriella ovata* (4)	FJ754026	1769	GQ246479	501	**JQ424856**	1855
***Diaxonella trimarginata***^a^**pop1** (3)	**JQ424833**	1767	**JQ424861**	551	**JQ424839**	1854
***Diaxonella trimarginata***^a^**pop2** (3)	**JQ424834**	1767	**JQ424860**	554	**JQ424837**	1843
*Metaurostylopsis cheni* pop1 (5)	GU170204	1767	GQ246481	486	**JQ424849**	1852
*Metaurostylopsis cheni* pop2 (6)	HM623916	1767	**JQ424863**	537	**JQ424854**	1852
*Metaurostylopsis cheni* pop3 (6)	FJ775720	1767	**JQ424862**	537	**JQ424843**	1851
***Metaurostylopsis struederkypkeae*** (3)	**JQ424832**	1768	**JQ424864**	538	**JQ424841**	1854
*Monocoronella carnea* (7)	FJ775726	1769	**JQ424870**	535	**JQ424855**	1855
*Neourostylopsis flavicana* (8)	FJ775718	1756	**JQ424869**	530	**JQ424857**	1844
*Nothoholosticha fasciola* (9)	FJ377548	1772	FJ858212	500	**JQ424846**	1848
*Pseudokeronopsis carnae* (10)	AY881633	1770	DQ503580	486	**JQ424836**	1852
*Pseudokeronopsis erythrina* (11)	FJ775723	1770	**JQ424868**	536	**JQ424852**	1852
*Pseudokeronopsis flava* (10)	AY881634	1770	DQ503579	484	**JQ424835**	1852
***Pseudokeronopsis* sp.** (3)	**JQ424830**	1770	**JQ424859**	534	**JQ424847**	1852
*Pseudourostyla cristata* (12)	FJ598608	1774	GQ246486	504	**JQ424848**	1851
*Thigmokeronopsis stoecki* (2)	EU220226	1771	GQ246485	480	**JQ424844**	1855
*Uroleptopsis citrina* (13)	FJ870094	1770	**JQ424858**	508	**JQ424850**	1852

References: 1 = Jiang et al. (2013); 2 = Yi et al. (2008b); 3 = present study; 4 = Liu et al. (2010); 5 = Chen et al. (2011c); 6 = Song et al. (2011); 7 = Chen et al. (2011b); 8 = Wang et al. (2011); 9 = Li et al. (2009); 10 = Yi et al. (2008a); 11 = Chen et al. (2011a); 12 = Chen et al. (2010b); 13 = Huang et al. (2010).

a A junior synonym of *D. pseudorubra* according to Berger (2006).

**Table 2:** Log likelihoods and *P*-values of AU (approximately unbiased) test for tree comparisons considering different topological scenarios. Significant differences (*P*-value < 0.05) between the best unconstrained and constrained topologies are in bold.

Datasets	Topology constraints	Log likelihood (-lnL)	AU (p)	Conclusion
SSU-rDNA	**Unconstrained**	12880.37762	0.980	–
	Monophyly of Pseudokeronopsidae^a^	13005.59089	**0.001**	**Rejected**
	Monophyly of Pseudourostylidae^b^	12903.10184	0.213	Not rejected
	Monophyly of *Pseudokeronopsis*	12927.9849	**0.017**	**Rejected**
	Monophyly of *Anteholosticha*	13242.98724	**7e−048**	**Rejected**
	Monophyly of *Metaurostylopsis*^c^	12924.05126	**0.002**	**Rejected**
	Monophyly of Holostichidae^d^	13312.33439	**2e−041**	**Rejected**
	Monophyly of Bakuellidae^e^	13333.85975	**8e−029**	**Rejected**
	Monophyly of Urostylidae^f^	13330.18477	**3e−033**	**Rejected**
ITS1-5.8S-ITS2	**Unconstrained**	5425.01386	0.995	–
	Monophyly of *Pseudokeronopsis*	5442.90804	0.063	Not rejected
	Monophyly of Pseudokeronopsidae^a^	5474.63174	**3e−018**	**Rejected**
	Monophyly of *Metaurostylopsis*	5467.50318	**0.001**	**Rejected**
	Monophyly of *Anteholosticha*	5501.2063	**9e-006**	**Rejected**
	Monophyly of Bakuellidae^g^	5482.78984	**0.003**	**Rejected**
	Monophyly of Urostylidae^h^	5450.91642	0.108	Not rejected
	Monophyly of core Urostyloidea	5435.138406	0.467	Not rejected
LSU-rDNA	**Unconstrained**	12873.84217	0.999	–
	Monophyly of *Pseudokeronopsis*	12906.45549	**0.003**	**Rejected**
	Monophyly of Pseudokeronopsidae^a^	13006.22066	**8e−062**	**Rejected**
	Monophyly of *Metaurostylopsis*	12976.50132	**2e−016**	**Rejected**
	Monophyly of Bakuellidae^g^	12992.20595	**2e−080**	**Rejected**
	Monophyly of Urostylidae^i^	12917.51593	**3e−004**	**Rejected**
Three genes	**Unconstrained**	25125.48866	1.000	–
	Monophyly of *Pseudokeronopsis*	25207.8366	**1e−156**	**Rejected**
	Monophyly of Pseudokeronopsidae^a^	25338.26538	**3e−004**	**Rejected**
	Monophyly of *Metaurostylopsis*	25282.48894	**3e−019**	**Rejected**
	Monophyly of Bakuellidae^g^	25328.42653	**4e−080**	**Rejected**
	Monophyly of Urostylidae^i^	25192.41704	**4e−058**	**Rejected**

a Pseudokeronopsidae: includes *Pseudokeronopsis*, *Nothoholosticha*, *Thigmokeronopsis*, *Apokeronopsis* and *Uroleptopsis*

b Pseudourostylidae: includes *Pseudourostyla* and *Hemicycliostyla*

c *Metaurostylopsis*: includes *Neourostylopsis flavicana*

d Holostichidae: includes *Holosticha*, *Psammomitra*, *Anteholosticha* and *Diaxonella*

e Bakuellidae: includes *Metaurostylopsis*, *Parabirojimia*, *Neobakuella* and *Apobakuella*

f Urostylidae: includes *Parabirojimia*, *Tunicothrix*, *Psammomitra*, *Holosticha*, *Metaurostylopsis*, *Diaxonella*, *Urostyla*, *Apourostylopsis*, *Thigmokeronopsis*, *Apokeronopsis* and *Anteholosticha*

g Bakuellidae: includes *Metaurostylopsis* and *Apobakuella*

h Urostylidae: includes *Metaurostylopsis*, *Diaxonella*, *Apobakuella*, *Urostyla*, *Anteholosticha*, *Thigmokeronopsis*, *Apokeronopsis* and *Neourostylopsis*

i Urostylidae: includes *Metaurostylopsis*, *Diaxonella*, *Apobakuella*, *Urostyla*, *Thigmokeronopsis*, *Apokeronopsis* and *Neourostylopsis*
